# Natural Biomaterials and Their Use as Bioinks for Printing Tissues

**DOI:** 10.3390/bioengineering8020027

**Published:** 2021-02-20

**Authors:** Claire Benwood, Josie Chrenek, Rebecca L. Kirsch, Nadia Z. Masri, Hannah Richards, Kyra Teetzen, Stephanie M. Willerth

**Affiliations:** 1 Department of Mechanical Engineering, University of Victoria, Victoria, BC V8P 5C2, Canada; cbenwood@uvic.ca; 2 Biomedical Engineering Program, University of Victoria, Victoria, BC V8P 5C2, Canada; josiec@live.ca (J.C.); hannahrichards@uvic.ca (H.R.); kyrateetzen3@gmail.com (K.T.); 3 Department of Chemistry, University of Victoria, Victoria, BC V8P 5C2, Canada; rebeccakirsch@uvic.ca; 4 Division of Medical Sciences, University of Victoria, Victoria, BC V8P 5C2, Canada; nadiam@uvic.ca

**Keywords:** bioink, 3D bioprinting, biomaterials, tissue engineering, regenerative medicine

## Abstract

The most prevalent form of bioprinting—extrusion bioprinting—can generate structures from a diverse range of materials and viscosities. It can create personalized tissues that aid in drug testing and cancer research when used in combination with natural bioinks. This paper reviews natural bioinks and their properties and functions in hard and soft tissue engineering applications. It discusses agarose, alginate, cellulose, chitosan, collagen, decellularized extracellular matrix, dextran, fibrin, gelatin, gellan gum, hyaluronic acid, Matrigel, and silk. Multi-component bioinks are considered as a way to address the shortfalls of individual biomaterials. The mechanical, rheological, and cross-linking properties along with the cytocompatibility, cell viability, and printability of the bioinks are detailed as well. Future avenues for research into natural bioinks are then presented.

## 1. Introduction

**Figure 1:**
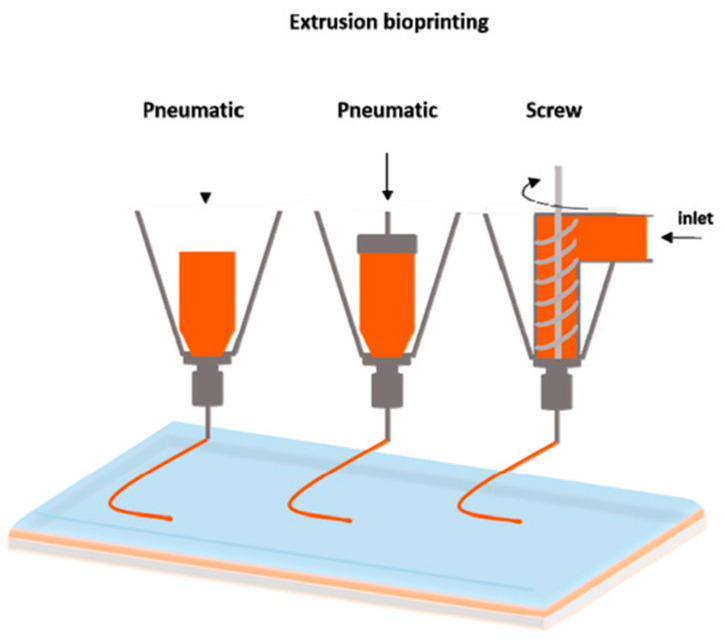
The main forms of extrusion printing [10]. This figure is being reprinted under a Creative Commons BY 4.0 license.

3D bioprinting uses additive manufacturing (objects are made layer-by-layer) to create objects that mimic biological constructs by patterning living cells and other biological materials [1]. Bioinks are the materials used to contain cells when bioprinting tissues. They provide structure for the bioprinted tissue and support and nutrients for the cells, creating an environment in which the cells can survive, grow, and proliferate. Many materials and combinations of materials can be used as bioinks: hydrogels (water-based gels) are the most popular and promising because they are biocompatible and have similar properties to the extracellular matrix (ECM) [2,3,4,5]. The ECM is the non-cellular scaffold secreted by the cells of all tissues and organs [6,7]. It supports cells and provides biochemical and biomechanical cues for several biological processes, including cell proliferation and differentiation [6,7]. This paper reviews the properties and applications of several natural bioinks that are suitable for extrusion printing. Extrusion printing is the most commonly employed method of bioprinting as it can be used with a wide range of compatible materials and their associated viscosities Figure 1 [8,9,10].

Bioprinting technology allows for control over various properties of the engineered object, such as its shape, size, and internal porosity [11]. The main bioprinting methods are extrusion, inkjet, laser-assisted, and stereolithography [11,12,13]. The advantages associated with extrusion printing include the ability to: (i) print one construct with multiple printheads and materials; (ii) print constructs with differences throughout (different cell types, densities, and signaling molecules); and (iii) print with higher cell densities than other methods [9]. Limitations of this type of printing include high shear stress and pressure, which reduce cell viability and functionality [9]. Another printing method is inkjet printing—a non-contact printing process (the printhead does not touch the object being printed) in which droplets of bioink are deposited onto a substrate or dish by a thermal actuator, piezoelectric actuator, or pressure-pulses [11,14]. Laser-assisted printing, based on the laser-induced forward transfer process, functions using energy from high-intensity lasers to deposit biomaterials [15]. Furthermore, stereolithography bioprinting uses digitally controlled light intensity to solidify a photo-sensitive material in a nozzle-free manner [16]. Many developed bioinks can be used for these printing methods, but a commonly cited challenge is creating suitable bioinks for different cells and applications since these often require specific bioink characteristics [17,18]. Additionally, these printing methods are similar to those used in other 3D printing applications for biomedical uses, including for 3D-printed prosthetics, customized surgical tools, and anatomical models [19,20,21]. However, these applications are beyond the scope of this review, which will focus on the use of bioprinting for tissue engineering purposes.

The 3D printing method dictates which properties the bioink must have before, during, and after gelation. Therefore, an effective bioink contains a combination of the following properties such as mechanical, including rheological, and crosslinking properties that are essential for shape fidelity and structural maintenance of the product, while cytocompatibility ensures the viability of the cells within the bioink [1,22]. The mechanical properties of a bioink can be tuned appropriately to ensure it meets strength requirements depending on the application. For example, load bearing organs like bone or cartilage will require a graft that demonstrates high mechanical strength [23]. Shear modulus and viscoelasticity are also important mechanical properties when considering various types of ECM [23]. For extrusion and droplet printing, shear thinning properties are required to compensate for the high shear stress developed during printing [24]. An important rheological property of bioinks is the viscosity; high viscosity allows the extruded filaments to maintain their shape before the crosslinking process that will set the print [25]. Additional factors to consider when evaluating the printability of a bioink are physicochemical factors like the swelling properties and gelation kinetics, both of which are unique to the material [22]. The swelling behavior of the hydrogel will determine the final shape and size of the 3D printed structure and the gelation kinetics, which are related to the crosslinking method, help the construct maintain its structure [26]. For example, gelation can be either physical or chemical depending on the crosslinking method and the desired interactions within the hydrogel.

Bioink hydrogels may be composed of natural biomaterials, synthetic materials, or a combination that highlights the favorable properties of both components. Natural biomaterials offer a favorable environment for cell growth by mimicking the natural ECM of tissues, self-assembling, and exhibiting biocompatibility and biodegradation properties [24,27]. However, they lack the mechanical properties required to maintain structural integrity within the in vivo microenvironment and can be unpredictable and unstable [24,28]. Poor mechanical properties can lead to difficulties in printing, less rigid tissue structures, and decreased support for the cells in the tissue [24,27]. Synthetic materials are controllable and can have photocrosslinking ability; however, they can be more cytotoxic than natural materials and, therefore, the environment they create may not promote the survival of cells [1,24]. For instance, the use of synthetic crosslinking agents can cause cytotoxic effects on cells that can be lessened with natural crosslinkers [29].

**Figure 2:**
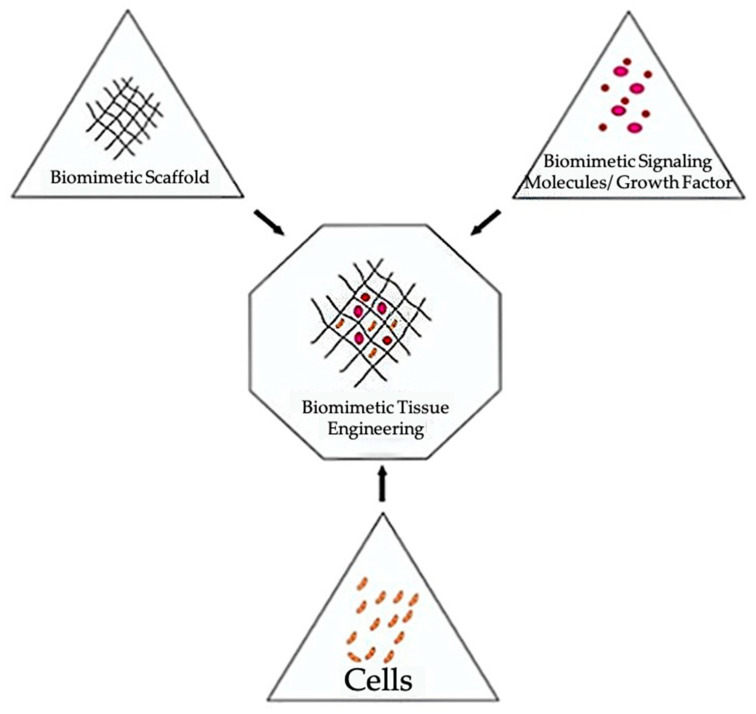
The main components used when constructing biomimetic tissues [32]. This figure is being reprinted under a Creative Commons BY 4.0 license.

Bioinks can be divided into two categories: scaffold-based and scaffold-free. Both bioink types can be utilized with extrusion printing. Scaffold-based bioinks incorporate cells into an exogenous biomaterial support structure Figure 2 [30,31,32]. This supporting scaffold is typically composed of hydrogels, microcarriers (porous, spherical structures to support cell growth and adhesion), or decellularized ECM (dECM) components [30]. The scaffold supports cell growth, proliferation, and differentiation while providing mechanical strength and biological and chemical cues to guide the assembly of a functional tissue [30,33]. Additionally, scaffolds are designed to degrade over time as the cells proliferate and begin to form the desired tissue. The scaffold is chosen in part based on its degradation rate, which should emulate the rate of ECM-formation by the cells incorporated into the construct [34]. Additionally, adjusting the rate of scaffold degradation can enable control over the release of growth and differentiation factors [34]. Although scaffold-based bioinks are designed to be highly biocompatible, challenges with these bioinks include host immunological responses during in vivo testing, material toxicity, disruption of cell-cell interactions, time requirements for scaffold degradation, and negative impacts of incompletely degraded scaffold on the mechanical properties of printed constructs [30,33,35,36,37].

In contrast, scaffold-free bioinks are composed solely of cells and their secreted matrices, without the need for supporting biomaterials [30,31,33]. These bioinks consist of cell aggregate structures, such as cell sheets, pellets, spheroids, or tissue strands, and rely on the ability of the cells to self-assemble into larger tissue constructs [30,31,34]. Scaffold-free bioinks eliminate the time requirements for scaffold degradation, reduce the need for extensive cell proliferation due to high initial seeding densities, minimize immunological responses in vivo, and improve cellular interactions and tissue biomimicry [33,34,35]. However, scaffold-based bioinks remain the most common due to their improved structural properties, reproducibility, scalability, and affordability [30,31,35]. Therefore, this review will focus on natural bioinks that belong to the scaffold-based category.

Natural bioinks have been applied to a diverse range of both soft and hard tissue engineering to create neural, cardiac, cartilage, vasculature, bone, and skin tissues [12,14,38,39,40,41]. Their ability to promote cell attachment and differentiation allow them to be used for drug testing and cancer research [42]. Personalized tissues can be created and, in the future, it may be possible to replicate whole organs for transplantation. The myriad combinations of the various bioinks described below allow for a wide range of functions.

## 2. Types of Natural Bioinks

A bioink should have and maintain similar properties to the targeted tissues, including: (i) physico-mechanical properties; and (ii) biological properties, to be considered biofunctional [24,43,44]. Not only does the bioink have to maintain cell viability, but it must also be printable. Accordingly, bioinks may be modified depending on the printer being used and the target tissue. For all these properties to be achieved, especially for extrusion-based bioprinting, a mix of two or more biomaterials is usually required [43,45]. Multicomponent bioinks are also often superior to those made up of solely one biomaterial as single component bioinks usually lack sufficient biocompatibility and high mechanical and functional requirements to form biomimicry tissues [45]. In addition, the components of multicomponent bioinks can complement one another, account for what the other bioink material might be lacking, and act as a supplementing element that can assist in the formation of more complex tissue constructs [43,45]. Furthermore, nanomaterials serve as an attractive addition to bioinks as they can lead to modifications, such as changes in bioink viscosity, and in some cases can make the bioink conductive, thus increasing signal transduction [44]. The following sections will discuss the properties of different natural bioinks, including agarose, alginate, gellan gum, dextran, hyaluronic acid (HA), silk, fibrin, collagen, dECM, Matrigel, cellulose, gelatin, and chitosan, and examples of common multicomponent bioinks and nanomaterials.

### 2.1. Agarose

Agarose is a natural polysaccharide derived from red seaweed that consists of repeating disaccharide units of D-galactose and 3,6-anhydro-L-galactopyranose [46,47]. Agarose is a part of the carbohydrate polymer family and is often used in tissue engineering applications due to its biocompatibility and thermo-reversible gelling mechanism [48]. When printed on its own, agarose is mainly used to help with mold formation when creating vascularized tissue constructs [33,46]. Norotte et al. used two printheads, with one dedicated to printing agarose rods to help control the wall thickness and diameter when creating tubular vascular grafts. The second printhead was used to deposit cell types, including fibroblasts and smooth muscle cells, into multicellular cylinders [33]. Agarose has also been used as part of a bioink designed to self-erode in order to form microchannels [49].

Agarose has a structure that resembles the ECM due to similar macromolecular properties [48]. Oxygen and other products can be diffused across its microstructure; therefore, it is often used as a component of bioinks to provide a support structure for cells [48]. Gu et al. created a bioink to print 3D neural mini-tissue constructs with agarose included in the ink specifically for structural support and to ensure the ideal viscosity of the bioink for printing [50]. Agarose hydrogels are also used to bioprint cartilage tissue [51]. Mesenchymal stem cell (MSC) laden agarose hydrogels were reinforced with 2% polycaprolactone (PCL) to increase stiffness [52]. This combination supports the production of hyaline cartilage and resulted in 80% cell viability after printing [52]. Native agarose does not react with other biological tissues and, therefore, it is often mixed with collagen, alginate, chitosan, or fibrin to increase its ability to support cell survival [47,48,53,54].

Carboxylated agarose is a derivative of native agarose with carboxylic acid groups on the polysaccharide backbone. Changing the degree of carboxylation causes an α-helix to β-sheet switch in secondary structure, allowing mechanical properties of the bioink to be modified without affecting the concentration [55,56]. Forget et al. bioprinted carboxylated agarose with human MSCs and achieved a 95% cell survival rate [57]. Gu et al. also created a carboxylated agarose-based bioink that had a high cell survival rate and was stiff enough to form 5–10 mm tall structures of various shapes without requiring extra support material [56]. Thus, agarose is a commonly used material for 3D printing applications.

### 2.2. Alginate

Alginate is a biocompatible anionic polymer derived from brown algae [58,59]. Alginates are block copolymers, and the exact sequence and ratio of a-L-guluronate and (1,4)-linked b-D-mannuronate residues depend on the alginate source [58,59]. Due to its biocompatibility and relatively low cost, alginate is commonly used for a variety of biomedical applications, including wound healing, drug delivery, and tissue engineering [58,59]. Alginate’s main advantage as a bioink is its ability to form hydrogels with properties similar to those of tissues’ ECM [58]. While mammals do not produce enzymes that cause alginate to degrade, other factors can impact its long-term stability in vivo. Alginate that is oxidized by periodate is prone to hydrolytic degradation, and ionically-crosslinked alginate gels erode in vivo due to divalent ions leaching into the media surrounding the gel [58,60]. Pure alginate also has low viscosity and zero shear viscosity, which impact its ability to retain its shape [61]. Alginate is unusual among natural bioinks in that it has very low bioactivity [61,62,63], which means that it does not support or promote cell proliferation. These barriers to using alginate as a bioink can be remedied by modifying it or mixing it with other materials. Adding nanocellulose has been shown to improve the rheological and mechanical properties and thus the printability of alginate-based bioinks [62]. Müller et al. added nanocellulose to alginate sulfate, which resulted in a bioink with improved rheological properties, a yield point instead of zero shear viscosity, and good day 28 cell viability [62]. Recently, Lee et al. found that adding methacrylated dECM to an alginate-based bioink improved the bioactivity [61]. Emami et al. explored oxidation as another method of stabilizing alginate bioinks [59]. Sodium periodate was used to oxidize sodium alginate, with different ratios of sodium alginate to sodium periodate resulting in different degrees of oxidation. Oxidizing the sodium alginate allowed its aldehyde groups to cross-link with the amine groups on gelatin, which resulted in a stable bioink with good cell adhesion, biocompatibility, and biodegradability properties [59]. Bioinks containing alginate have applications in a variety of areas of tissue engineering, including the development of many types of bioprinted tissues, drug delivery, and wound treatment [58,59,61,62,64,65].

### 2.3. Cellulose

Cellulose serves as the primary structural material in plant cell walls due to its rigid structure [66]. It is a polysaccharide made up of (1–4) linked β-D-glucopyranosyl units [67]. Carboxymethyl cellulose (CMC), a cellulose ether that is water soluble, can be used to modify the viscosity of other polymers with less ideal rheologic properties [68]. In one study that created bone tissue constructs, CMC was combined with a poly(lactic-co-glycolic acid) bioink to create the ideal viscosity for deposition – the highest viscosity that would not obstruct the syringe tips. The addition of CMC also allowed for cells to be included in the ink and printed successfully [69]. Janarthanan et al. mixed CMC with glycol chitosan hydrogels to create a gel-based ink that had both stability and cell compatibility. The CMC provided reinforcement to the hydrogels, helping with the stability and shape fidelity of the final constructs [68,70].

Cellulose nanocrystals occur when the cellulose chains are highly ordered and can promote mechanical strength along with shear thinning behavior [71]. They are incorporated into many different bioinks, improving the elasticity, strength, and porosity of the constructs created, and when blended with other materials can also improve the viscosity of bioinks [72,73]. Jiang et al. created a bioink that incorporated cellulose nanocrystals into a bioink made of oxidized dextran and gelatin hydrogels. The CNC helped to improve the porosity of the constructs created [72]. Along with being biocompatible, nanocellulose does not enable bacterial growth, making it an attractive option for wound dressing applications [73]. Another study utilized the shear thinning of nanofibrillated cellulose and combined it with alginate in order to bioprint cartilage tissues. Human chondrocytes were successfully included in the ink and after 7 days had a cell viability of 86% [74]. These studies demonstrate the benefits of incorporating cellulose into a bioink blend.

### 2.4. Chitosan

Chitosan is a naturally derived polysaccharide that is made through chitin deacetylation. Chitosan is typically poorly soluble in water but can be dissolved in solutions with a pH of 6.2 or lower [75,76]. Furthermore, chitosan is nontoxic, biodegradable, biocompatible, bio-adhesive, and renewable [75,76]. However, chitosan has weak mechanical strength, which limits its use for creating hard tissues like cartilage [75].

He et al. modified chitosan with ethylenediaminetetraacetic acid (EDTA) before the addition of Ca^2+^ to increase the amount of chitosan-Ca^2+^ crosslinking, which enhanced the stability and mechanical properties of chitosan for chondrocyte support [75]. Varying concentrations of the two bioink components, chitosan and modified chitosan, resulted in altered printability and gelation abilities, and higher proportions of modified chitosan resulted in higher storage and loss moduli. Modified chitosan was the main component that contributed to strength enhancement. Furthermore, their bioink was analyzed and found to have low cytotoxicity, no effect of the hydrogel mesh on chondrocyte toxicity, no impedance on cell proliferation, fast gelation, high precision during printing, and the ability to tune mechanical strength and viscoelastic properties through the adjustment of the two component proportions [75]. In another study, a chitosan bioink was prepared by dissolving chitosan in an acidic mixture and its properties were analyzed for extrusion printing [76]. Concentrations of chitosan ink higher than 11 wt% and lower than 4 wt% were found to be too viscous and too dilute, respectively, while an optimal viscosity was found from a median concentration. The printed structures had high resolution (30 µm) and high shape retention. Good mechanical properties of the chitosan hydrogel (high max strength break of a neutralized filament was ~97 MPa in dry state, and high strain break at ~360% in a wet state) were found [76]. In conclusion, chitosan offers many advantages to bioprinting but often requires additional components to improve its mechanical strength.

### 2.5. Collagen

Collagens are the most prevalent proteins in mammals, comprising approximately 30% of the total mammalian protein mass [77]. They are hydrophilic proteins that are important structural components of the ECM [77,78]. Collagens consist of three polypeptide chains, known as α chains, and contain triple helical domains [77]. There are 28 different types of collagen, which are composed of varying quantities of triple helices and different combinations of α chains [77,78]. Collagens do not cause significant immunological responses and have integrin-binding domains, which promote cell adhesion, attachment, and growth [30]. However, the immunogenicity of collagen can be affected by the presence of other proteins, cell remnants, and crosslinking reagents, and animal-derived collagen may lead to inflammation and disease transmission [79,80]. Collagen type I is a member of the fibril-forming subfamily of collagens [78] and is commonly used in bioprinting [30]. However, it is not often used as a bioink on its own due to its mechanical instability and slow gelation rate at physiological temperatures, which limit its ability to hold its shape once extruded [30]. Collagen maintains a liquid state below 37 °C [30,81].

Studies using collagen alone as a bioink often aim to improve its mechanical properties by using sacrificial supports (temporary materials that maintain the structure of bioprinted constructs but are removed post-printing) [82,83] or by directly modifying characteristics of the collagen bioink, such as the concentration or crosslinking method utilized [84,85,86]. Several studies have also investigated methods to improve the printability of collagen by controlling the gelation kinetics and storage modulus of collagen bioinks [84,86,87]. An increased storage modulus, particularly one that significantly exceeds the loss modulus, has been found to correspond to improved printability of collagen [86]. Additionally, Diamantides et al. showed that both gelation kinetics and the storage modulus of collagen bioinks are dependent upon pH levels [84]. The average storage modulus after complete gelation was found to be highest at pH values of 7.5–8.0 and 8.0–8.5, but decreased outside of these ranges [84]. In this study, a blue light activated riboflavin crosslinker was also shown to increase the storage modulus of the collagen [84]. However, the riboflavin crosslinking resulted in an approximately 20% reduction in chondrocyte viability [84]. In a more recent study, it was found that the storage modulus of a type I collagen bioink was dependent on both the seeding density of cells and the degree of gelation [87]. Although the collagen maintained high levels of printability and chondrocyte viability across a range of cell densities, the storage modulus was found to increase with cell density before gelation but decreased with higher cell density after gelation [87].

Collagen is often combined with other biomaterials to improve the structural integrity, printability, and bioactive properties of natural bioinks. For instance, Yang et al. found that adding type I collagen to an alginate-based bioink improved its mechanical strength, helped preserve chondrocyte phenotypes, and suppressed undesired differentiation when printing cartilage constructs [51]. Similarly, the applicability of collagen-alginate composite bioinks for bioprinting of cartilage was demonstrated in a study by Liu et al., which showed that printed collagen-alginate hydrogels could support sustained drug release from incorporated PCL microspheres [88]. A recent study also examined the rheological properties of collagen-chitosan composite bioinks with different component ratios [89]. Collagen-chitosan blends exhibited shear-thinning behavior and negligible cytotoxicity effects on NIH-3T3 fibroblasts, which are encouraging properties for future work with this biomaterial combination [89]. Overall, extensive work has been done to optimize collagen as both a stand-alone bioink and as part of multicomponent bioinks.

### 2.6. Decellularized Extracellular Matrix

dECM is obtained through decellularization of tissues using a variety of physical and chemical methods, including freeze-thaw cycles, detergents, or enzymatic agents [90]. Although a certain amount of disruption to the ECM is unavoidable during this process, tissue decellularization aims to remove all cellular components of the tissue while maintaining as much of the structure and composition of the ECM as possible [90]. Retaining the native structure of the ECM offers several benefits for use as a bioink material, including potential elimination of the need for crosslinker [91] and the ability to induce tissue-specific characteristics into printed constructs through choosing the tissue source of the dECM [7,91,92]. For instance, Han et al. tested four porcine-derived dECM bioinks from different sources (liver, heart, skin, and cornea) and demonstrated that the tissue source of the dECM generated tissue-specific gene expression in human bone marrow MSCs [92]. dECM is also one of the few natural biomaterials that is commonly used as a bioink on its own. dECM bioinks for extrusion printing have been produced using a variety of tissue sources, including the heart [91,92,93,94,95,96], skin [92,97,98,99], liver [92,100], intestines [101], cornea [92,102], bones [103], and tendons [104,105].

dECM has been significantly characterized for cardiac applications. Decellularized heart tissue has been shown to support the maturation, differentiation, and viability of cardiomyocytes [93,94]. Shin et al. demonstrated that incorporating Laponite-XLG nanoclay and poly(ethylene glycol) diacrylate (PEGDA) for photopolymerization could improve the structural fidelity of heart dECM bioinks laden with hiPSC-derived cardiomyocytes while maintaining over 94% viability after seven days [94]. Changing the concentration of PEGDA enabled tuning of the compressive Young’s (or elastic) modulus, which can allow for better replication of the mechanical properties of both normal and fibrotic heart tissue [94]. Additionally, heart dECM bioinks have shown promise for printing human cardiac progenitor cells [95,96]. Jang et al. printed pre-vascularized stem cell patches composed of a heart dECM bioink laden with cardiac progenitor cells and MSCs [96]. In vivo testing in mice demonstrated that patches patterned with the two cell types promoted vascularization, maintained cell viability, and decreased cardiac remodeling and fibrosis [96].

Additionally, dECM bioinks have been validated for skin tissue engineering, particularly for wound healing applications. For instance, Kim et al. printed a pre-vascularized skin patch using skin-derived dECM laden with endothelial progenitor cells and adipose-derived stem cells, which promoted wound closure, epithelialization, neovascularization, and blood flow during in vivo testing in mice [97]. In vitro analysis also showed that the dECM constructs shrunk less than collagen-based constructs [97]. Similarly, combining skin-derived dECM with a fibrinogen-based bioink was recently found to improve the mechanical properties and viability of a bioprinted skin model incorporated with primary human skin fibroblasts [98]. Collagen from the dECM improved the structural fidelity of the fibrinogen constructs and SEM imaging showed that the dECM and fibrinogen combination produced micro-architecture that resembled human skin [98]. These studies exemplify the beneficial properties of dECM bioinks for bioprinting a variety of tissues.

### 2.7. Dextran

Dextran is a natural, nontoxic, hydrophilic homopolysaccharide consisting mostly of α-1,6-linked D-glucopyranose residues [44,106]. Because dextran only contains hydroxyl groups, which do not provide support for cell attachment, it is common to chemically modify dextran to allow for functional affinity binding sites [106]. An advantage of dextran is that it can be used to create biodegradable scaffolds due to its degradation by dextranase, which is an enzyme naturally found in mammals [106].

Dextran is not typically used as a bioink by itself because of its poor mechanical strength, so it is often combined with other natural biomaterials [107]. Notably, oxidized dextran has been shown to act as a natural crosslinker for gelatin-based bioinks, resulting in improved printability and structural fidelity of extruded structures [72,108,109,110]. Pescosolido et al. utilized photocrosslinkable hydroxyethyl-methacrylate-derivatized dextran (dex-HEMA), a dextran derivative, to improve the stability of 3D-bioprinted HA hydrogels [111]. In this study, a semi-interpenetrating network scaffold of HA and dex-HEMA was printed and demonstrated high porosity, shear thinning at shear rates above 0.05 s^−1^, and good structural integrity while maintaining the viability of encapsulated equine chondrocytes for three days [111]. Additionally, Harman et al. demonstrated that a bioink composed of the synthetic polymer poly(ethylene-3,4-dioxythiophene) and dextran sulfate could be extrusion printed and could support the viability, morphology, and proliferation of fibroblasts when added to cell culture medium [112]. The conductive properties of this composite bioink could enable future applications involving the electrostimulation of cells [112].

### 2.8. Fibrin

Fibrinogen is a soluble protein found in blood and the enzyme thrombin catalyzes the digestion of fibrinogen into fibrin monomers. Fibrin is an insoluble, biocompatible, and biodegradable biopolymer with properties that can be adjusted by modifying the concentrations of both the thrombin and fibrinogen present [113]. Fibrin can also be blended with other materials such as PCL to adjust its properties [113]. This allows fibrin properties to imitate both hard and soft tissues. Fibrin is also a popular choice for bioinks because it allows for communication between cells due to its non-linear elasticity [113]. Fibrin-based bioinks are used to create a wide range of tissues including neural, cardiac, skin, and vascularized tissues [12,39,41,42,114]. Fibrin based bioink printed with Aspect Biosystems novel RX1 bioprinter showed cell viability levels of neural progenitor cells (NPCs) to be greater than 81% [115]. Sharma et al. combined this bioink with guggulsterone releasing microspheres in order to differentiate human induced pluripotent stem cell (hiPSC)-derived NPCs into neural tissues consisting of dopaminergic neurons [116]. Fibrin is a viscoelastic polymer that is often not printed as a stand-alone material due to its high viscosity that makes extrusion of the bioink challenging in its cross-linked form [117]. A lack of shear thinning behavior in fibrin does not allow for the decrease in viscosity required for the successful extrusion of bioink. Its pre-polymer form, fibrinogen, is also difficult to print alone because it is unable to maintain its shape [113].

These challenges in printing fibrin have been overcome by utilizing numerous different strategies, the first of which is crosslinking. Lee et al. created a tumor model to analyze a novel glioblastoma treatment. A fibrin-based bioink was printed with a crosslinker to polymerize the bioink as it was extruded from the microfluidic printhead [42]. Smits et al. utilized this same method and bioink to evaluate the effectiveness of Compound 15 in treating glioblastoma multiforme [118]. In a different study, both a bioink and crosslinker were extruded from two different needles to create a core-shell design [119]. The second strategy for printing fibrin is to use a support bath that helps to maintain the shape of the bioprinted construct [64]. Freeform reversible embedding of suspended hydrogels (FRESH) —a novel method of bioprinting used by Hinton et al.—was used to extrude a fibrinogen ink into a gelatin support bath containing thrombin that was then removed after printing to create bifurcated tubes [120]. Finally, the method of combining fibrinogen with materials that have more viscous properties, such as gelatin, was used to bioprint cardiomyocyte-laden constructs [121].

### 2.9. Gelatin

Gelatin, a natural polymer created through collagen hydrolysis, has been widely used as a bioink component [122]. Gelatin can form hydrogels after being cooled at low temperatures (20–30 °C) and is thermo-sensitive, meaning its bonds are easily broken by heat, resulting in its ability to be printed and stacked on itself in a controlled fashion [123]. Gelatin provides good biocompatibility, solubility, and degradability to bioinks [122]. As well, the viscosity of gelatin-based bioinks can be easily changed by altering the temperature or concentration of gelatin within the bioink. This is advantageous since certain viscosities are not compatible with extrusion bioprinting. Gelatin also has a number of side chains that allow for its chemical crosslinking and modification [122,124].

Gelatin has been combined with other natural or synthetic materials when manufacturing bioinks due to its many advantages [107,108,125]. For example, its quick gelation at moderate temperatures gives printed constructs strong initial stability even when printed with other, less stable materials [126]. Afterwards, when in physiological conditions, the gelatin will dissolve, leaving the other biomaterial (e.g., silk) behind [126]. Unmodified gelatin does not crosslink alone and requires chemical reactions (e.g., with N,N-(3-dimethylaminopropyl)-N’-ethyl carbodiimide and N-hydroxysuccinimide) or additional components to be added (e.g., alginate, chitosan, fibrinogen, hyaluronan) to instigate crosslinking [123,127]. Berg et al. optimized a gelatin/alginate/Matrigel bioink to be used as a scaffold for human alveolar A549 cells [126]. When testing combinations using 1–10% (w/v) gelatin, it was reported that low amounts of gelation resulted in insufficient shape fidelity, but too high amounts resulted in unprintable hydrogels. A 2% (w/v) alginate and 3% (w/v) gelatin bioink was determined to be the best combination. When printing A549 cell-laden bioink, Matrigel was added to improve the bioink biocompatibility and expediate gelation.

Gelatin is often used in the form of gelatin-methacryloyl (GelMA), which is created through a gelatin and methacrylic anhydride reaction [122]. GelMA can be used for a broad range of tissue engineering applications, and is especially suitable for the production of load-bearing tissues like bone, cartilage, skin, and vascular networks [122]. A benefit of GelMA is that it does not require crosslinking agents or localized gelation during extrusion printing [122], but it does require the use of a photoinitiator followed by UV light exposure, which can cause decreased cell viability [128]. As well, high concentrations of GelMA (>7%) result in viability and biocompatibility deficits [129].

Liu et al. used a low concentration of GelMA (3%) and a cooling process to develop soft (~1.8 kPa) cell-laden constructs with high shape fidelity [129]. The GelMA bioink had self-healing (at concentrations of 3% and 4%) and shear-thinning properties. It was determined that higher GelMA concentrations and lower temperatures resulted in faster gelation speed, but lower concentrations resulted in more porous and flexible constructs [129]. In general, gelatin and its derivative, GelMA, are some of the most common natural bioinks used because of their thermo-sensitive capabilities and advantages this gives in altering viscosities.

### 2.10. Gellan Gum

Gellan gum is a linear, anionic polysaccharide secreted by the bacterium Pseudomonas elodea as a product of fermentation [130,131]. Gellan has a repeating pattern of one L-rhamnose, one D-glucuronic acid, and two D-glucose subunits [2,130]. It is biocompatible, biodegradable, and non-toxic with good mechanical properties and high gelling efficiency [2,130]. In the field of tissue engineering, gellan gum’s primary applications are in bioprinting cartilage [3,4], skeletal tissue [132], and brain-like structures [133]. Although the main component of cartilage is collagen, gellan gum is added to collagen derivatives, such as GelMA, to improve the viscosity and increase the yield stress of the ink [4].

When gellan gum’s biocompatibility is desired but stronger mechanical properties are necessary, gellan gum has successfully been added to a variety of other components. Hu et al. have shown that mixing gellan gum and PEGDA creates a bioink with good mechanical and rheological properties and high cell viability [2,3]. In one experiment, various structures were printed, using two automatic switching printheads (one filled with gellan gum and PEGDA, and one with poly(lactic acid) wire). After the structures were exposed to UV radiation to create scaffolds, their properties were tested. A rheometer tested the rheological properties, a universal testing machine was used for a compression test, a sodium hydroxide bath was used to test degradation, and the cell viability was assessed by live-dead staining [3]. After cross-linking, the bioink was able to withstand a shear rate of > 300 s^−1^, and formed a strong and stretchable structure with a high cell proliferation rate and cell viability above 90% during the culture time [3]. Chen et al. also found that combining gellan gum, sodium alginate, and a thixotropic magnesium phosphate-based gel to increase bioactivity produced a hybrid bioink with good gelation, mechanical, rheological, and printing properties that promoted cell proliferation and survival [5]. Cartilage test structures, such as human ears and noses, were printed, cross-linked in a calcium chloride solution, and tested using similar methods to those carried out by Hu et al. [3,5]. All of the combination bioinks exhibited low viscosities and high shearing rates, [5], both of which improve printability. Additionally, the constructs with higher concentrations of gellan gum and lower concentrations of sodium alginate had better cell proliferation [5]. These two experiments illustrate gellan gum’s ability to promote cell proliferation and improve the rheological properties of combination bioinks.

### 2.11. Hyaluronic Acid

Found in the ECM, HA naturally occurs in the ECM of mammalian tissues, including most organs and tissues of the central nervous system (CNS) [111,134,135,136]. HA is biocompatible, biodegradable, and bioresorbable, meaning it can be left in the body where it may dissolve or be absorbed [135]. It also has high porosity, which allows for easy diffusion of nutrients and waste products. HA has the ability to maintain a hydrated environment, making it an ideal tool for promoting wound healing [111,135]. The applications of HA extend from CNS and brain tissue engineering to bone and cartilage tissue engineering. It can be applied to organs and connective tissue and can be used as a space-filling scaffold [111,135,136,137,138,139,140]. HA lacks the mechanical integrity to function as an independent bioink; it has low stability caused by its high water solubility [111,141]. In addition, cells do not stick to the surface of HA, so it must be mixed with other components to promote cell adhesion [136]. The drawbacks of HA are often resolved by crosslinking and combining it with other components, as discussed in the following paragraphs, to form a hydrogel suitable for bioprinting [111,135,137,138,140,141].

Hou et al. developed a HA hydrogel modified with laminin, a component in the ECM that assists with cell adhesion and improves mechanical properties [139]. Laminin was immobilized on the backbone of the cross-linked HA and this hydrogel was implanted into cortical defects created in rats. After 6 and 12 weeks, the results showed that the hydrogel created a scaffold to foster infiltration, angiogenesis, and neurite extension while inhibiting glial scar formation. While this study did not test the hydrogel for bioprinting applications, it demonstrates a possible HA combination that exhibits advanced wound healing and mechanical properties.

Mazzocchi et al. hypothesized that methacrylated collagen I and thiolated HA could form a hydrogel with suitable properties for extrusion bioprinting [140]. A hydrogel composed of 3:1 collagen I: HA was ideal to bioprint a liver microenvironment due to the bioink’s mechanical properties, printability, and high cellular viability. The liver model contained human hepatocytes and liver stellate cells; it was maintained for two weeks to demonstrate that the extrusion printing method did not adversely affect the cells. This study established a simple bioink that can be extrusion bioprinted and can be improved upon by the addition of ECM components like laminin or fibronectin. This bioink has the potential to provide a platform for the biofabrication of various types of tissue for human use.

### 2.12. Matrigel

Matrigel consists of a composite, gelatinous mixture extracted from mice tumors that mimics the human extracellular matrix (hECM) and contains proteins like laminin, collagen, and entactin [142,143,144]. This protein-based biomaterial provides a microenvironment for cells to grow in since it contains peptides and growth factors that help with cell growth and adhesion, components that are lacking from polysaccharide-based hydrogels [145]. Matrigel crosslinks at room temperature and, thus, needs a temperature-controlled system when using it for extrusion printing [144]. Matrigel has poor mechanical strength, like most protein-based hydrogels, so it is not ideal for printing [145].

Li et al. made a sodium alginate-Matrigel (SA-MA) hydrogel to print a 3D scaffold for assistance with the neural differentiation of ectomesenchymal stem cells in vitro and compared its properties to SA-hyaluronic acid and SA-gelatin hydrogels [146]. CaCl_2_ was used as a crosslinking agent [146]. SA-MA and SA-gelation had the shortest gelation time, and SA-MA was the most suitable combination for complete gelation. Viscosity was altered by changing alginate and Matrigel concentrations, with 0.5/30 alginate/Matrigel concentration being the most suitable for printing and molding. The SA-MA hydrogel was the most suitable for cell growth and had the highest cell viability (88.22 ± 1.13%) of the hydrogels. Finally, increasing the Matrigel ratio of the bioink resulted in decreased scaffold degradability [146]. In another study, a collagen-Matrigel combination was used to print breast cancer cell-laden networks [143]. The addition of Matrigel allowed for the use of a lower collagen concentration (0.8 mg/mL) that more accurately represented collagen fiber networks used during cell culturing in previous studies. Matrigel can affect the morphology of the collagen fibers, as lower concentrations of Matrigel resulted in longer collagen fibers, although varying the Matrigel concentrations did not affect the collagen fiber alignment [143]. Although in vivo applications of Matrigel are limited due to its derivation from murine tumors, Matrigel bioinks present numerous opportunities for ex vivo research, including drug screening, 3D cell culturing, and cancer modelling applications [147,148].

### 2.13. Silk

Silk is found abundantly in nature, either from silkworms or spiders, and it has many diverse applications [149,150]. Silk fibroin (SF) is useful in tissue engineering due to its mechanical properties, biocompatibility, and controllable degradability [125,151,152]. This natural fibrous polymer has shear thinning properties that make it ideal for extrusion bioprinting [153]. SF is an attractive natural hydrogel component because it can accommodate chemical interactions other than covalent bonds, meaning it can be physically crosslinked, which removes the need for harsh crosslinking chemicals [125]. The disadvantages of SF include low viscosity and frequent clogging during printing [125].

Singh et al. developed a crosslinker-free hydrogel composed of silk and gelatin, where gelatin was used as a bulking agent [125]. These two components interacted by entanglement and physical crosslinking, eliminating the need for a crosslinking agent. A 3D bioprinted chondrocyte-laden construct of a human ear was printed and demonstrated good print fidelity of anatomical structures. The structure demonstrated in vitro and in vivo biocompatibility and provided promising advancements in cartilage tissue engineering. Rodriguez et al. aimed at producing a bioink to aid in soft tissue regeneration composed of SF, gelatin, and glycerol to induce physical crosslinking [153]. Irregular shapes, such as a human cheek segment, were printed and implanted in a mouse model. The study concluded that the system’s rheology could be tuned by tuning the component ratios and concentrations to obtain different physical and mechanical properties. In addition, the biocompatible material maintained its shape for up to 3 months while promoting tissue integration.

## 3. Directions for Future Work and Conclusions

Future work in developing natural biomaterials for use in bioinks for tissue engineering aims to create more complex shapes, vasculature, and functional tissue structures. This can be achieved by optimizing the combination of biomaterials used for better mechanical, rheological, and biological properties. Optimizing and developing novel printing methods will also help to create larger and functioning organ structures. A proof of concept for this is FRESH, an innovative printing method used to print a human heart along with a functional tri-leaflet heart valve composed of collagen [82]. Additionally, improving the speed and resolution of printing is another goal of innovations in printing methods. For instance, Cellink and Prellis Biologics have introduced the Holograph X bioprinter, which utilizes holograms produced by spatial light modulators [154]. Using multiple printheads and materials to create complex structures can also be investigated. Future work involving adding drug-loaded microspheres to bioinks could increase cell viability along with stem cell differentiation and survival [116]. Incorporating drug laden microspheres into bioprinted constructs is one example of 4D bioprinting. 4D bioprinting is an extension of 3D bioprinting in which a fourth dimension—time—leads to a change in shape, functionality, or biophysical characteristics of printed constructs due to internal or external stimuli [155,156]. Physical, chemical, or biological stimuli can be utilized in 4D bioprinting, including temperature changes, introduction of electric fields, pH changes, and enzymes [156]. Further investigating 4D bioprinting methods could lead to improved ability to produce structures that are difficult to obtain using traditional bioprinting methods, such as hollow tubes required for vasculature [157]. In addition, a co-culture construct was developed to imitate a brain and bioprinting was used to bioprint neurospheroid-laden designs [158]. This model could be expanded to explore the potential of using two or more multicomponent bioinks with or without the same crosslinker that can each support a different cell type. This would replicate a co-culture and allow the cells to thrive in a well-suited environment. Future work should investigate optimizing scaffold-free bioinks, which are composed only of cells without supporting biomaterials [30,31,33]. This should involve investigating methods of improving the structural integrity, reproducibility, and scalability of scaffold-free bioinks [35]. Transitioning towards bioinks consisting exclusively of cells could mitigate issues associated with scaffold-based bioinks, such as material toxicity, interruption of cell interactions, and impacts of scaffold degradation on mechanical properties [35]. Ultimately, scaffold-free bioinks could reduce immune responses in vivo and improve the biomimicry of bioprinted tissues [33,35]. Further challenges to be overcome include the scaling up of manufacturing bioink and its standardization.

## Data Availability

Not applicable.
